# Can the Fact That Myelin Proteins Are Old and Break down Explain the Origin of Multiple Sclerosis in Some People?

**DOI:** 10.3390/jcm7090281

**Published:** 2018-09-14

**Authors:** Roger J. W. Truscott, Michael G. Friedrich

**Affiliations:** Illawarra Health and Medical Research Institute, University of Wollongong, Wollongong, NSW 2522, Australia; michaelf@uow.edu.au

**Keywords:** proteomics, age-related, isoaspartate, posttranslational modification, protein decomposition, myelin basic protein

## Abstract

Recent discoveries may change the way that multiple sclerosis (MS) is viewed, particularly with regard to the reasons for the untoward immune response. The fact that myelin proteins are long-lived, and that by the time we are adults, they are extensively degraded, alters our perspective on the reasons for the onset of autoimmunity and the origin of MS. For example, myelin basic protein (MBP) from every human brain past the age of 20 years, is so greatly modified, that it is effectively a different protein from the one that was laid down in childhood. Since only a subset of people with such degraded MBP develop MS, a focus on understanding the mechanism of immune responses to central nervous system (CNS) antigens and cerebral immune tolerance appear to be worthwhile avenues to explore. In accord with this, it will be productive to examine why all people, whose brains contain large quantities of a “foreign antigen”, do not develop MS. Importantly for the potential causation of MS, MBP from MS patients breaks down differently from the MBP in aged controls. If the novel structures formed in these MS-specific regions are particularly antigenic, it could help explain the origin of MS. If verified, these findings could provide an avenue for the rational synthesis of drugs to prevent and treat MS.

## 1. Introduction

Despite many years of research, the initiating events that lead to multiple sclerosis (MS) remain a mystery. Several genes have been linked to a higher likelihood of developing MS, in particular, those of the human leukocyte antigen (HLA) system. A consistent finding is the association between MS and DR15 and DQ6 alleles of the major histocompatibility complex. Genome-wide association studies have uncovered over 200 other genes outside of the HLA locus that increase the probability of MS to a small extent [1,2].

Environmental contributors, such as infections, have been difficult to link directly with the onset of MS. The role of microbiota is becoming increasingly of interest following the discovery that microbes can trigger chronic immune disorders to dietary antigens, e.g., celiac disease [3], in addition to affecting the outcome of neurological diseases [4]. In a remarkable linkage between cellular and microbiological strands, it has been shown recently that tryptophan metabolites derived from gut bacteria can enter the CNS where they act on transcription factors present in microglia and astrocytes [5]. In this way, the gut can modulate inflammation in the CNS and affect the outcome of experimental autoimmune encephalomyelitis (EAE), a common animal model of MS.

## 2. Long-Lived Proteins and Autoimmunity

The reasons for developing autoimmunity in general, and MS in particular, are not well understood. One feature that has been largely overlooked is the role of endogenous antigen modification in triggering autoimmunity. In the bodies of adults, long-lived proteins are ubiquitous [6,7,8]. In early life, such long-lived proteins are recognised as “self” however, over time, they breakdown and this decomposition effectively creates new proteins that may be recognised by the body as being “foreign”. The subject of this review are the structural changes to long-lived proteins that occur inevitably with age. If these alterations are significant, new antigens are effectively created within the body. Such processes may have particular relevance for the induction of MS, since myelin proteins are long-lived proteins.

**Figure 1:**
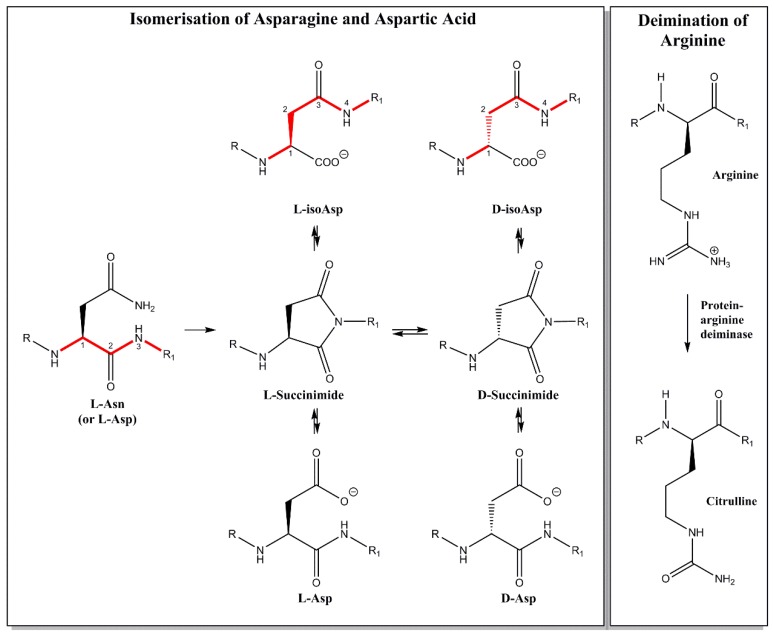
When old proteins in the body degrade, novel structures are formed. l-Asp and l-Asn residues can spontaneously convert over time to 4 isomeric forms, via a cyclic intermediate as illustrated. An uncharged Asn becomes a mixture of four negatively charged Asp residues. Probably of greatest impact as potential immune inducers are l-isoAsp and d-isoAsp. In the case of d-isoAsp the α-carbon is racemised and the polypeptide chain is also extended by the insertion of a CH_2_ group, as highlighted by the red bonds. In adult MBP, many positively charged Arg residues have been converted to neutral citrulline residues. Together these abundant post translational modifications (PTMs) lead to protein unfolding and the generation of novel antigens.

Over time, long-lived proteins undergo a plethora of modifications, such as racemisation, truncation, deamidation, deimination, and cross-linking (reviewed in Ref. [9]) with each single modification potentially being sufficient to break immune tolerance. Age-related modification of proteins at a number of sites in the body may underlie the observation that the levels of autoantibodies increase as a function of age [10]. In the case of another autoimmune condition, systemic lupus erythematosus, [11], Mamula et al. have proposed a role for isoaspartate (isoAsp), an isomer of Asp that is formed spontaneously in old proteins, [12] (see Figure 1) in generating the autoantibodies to histones; another family of long-lived proteins.

## 3. Long-Lived Proteins and Multiple Sclerosis

Amongst the list of long-lived proteins are the structural proteins of myelin [7,9]. Indeed, these may not turn over at all. Evidence for myelin proteins being long-lived comes from two main sources. Firstly, stable-isotope labelling revealed little or no turnover [6]. Secondly, the degree of amino acid racemisation of human MBP as a function of age [12] matched closely that of crystallin proteins from the lens that are well-known not to be replaced during our lifetime [7]. Once the myelin sheath is laid down in childhood, the major proteins within it, such as myelin basic protein (MBP), may be with us for all of our lives. Although this is, in many ways, a remarkable finding, why does this matter, and how could this play a role in the onset of human MS?

It turns out that proteins are not stable in a biological environment. Over time they break down. To a large degree it seems that decomposition is largely non-enzymatic and that old proteins decay due to the intrinsic instability of certain amino acid residues. Some reactions have been known for years. For example, aspartic acid and asparagine racemise i.e., they change from the l-form to the d-isomer, as well as converting to a different isomeric form, isoAsp (Figure 1) [13]. 

Other spontaneous reactions of old proteins include crosslinking [14,15], deamidation [16], and peptide bond cleavage [17]. It should be noted that not all modifications to long-lived proteins are spontaneous, for example, deimination of arginine residues has been attributed to the action of protein arginine deiminase (Figure 1).

The many novel modifications of old proteins, and their time courses as a function of age [16,18], have been documented in the human lens, where it is well-established that proteins do not turn over [8]. It is of interest that another age-related disease, cataract, can be traced to a greater degree of protein modification at specific sites in lens crystallins [19,20]. If these reactions are extensive, the structure and properties of the affected long-lived proteins change dramatically. This may have significant consequences, for example, it is possible that the body may no longer recognise the altered protein as being “self”. If this occurs, an immune response could be mounted against the modified old protein. These subjects have been summarised in a recent review [9].

## 4. Myelin Basic Protein and Age

**Figure 2:**
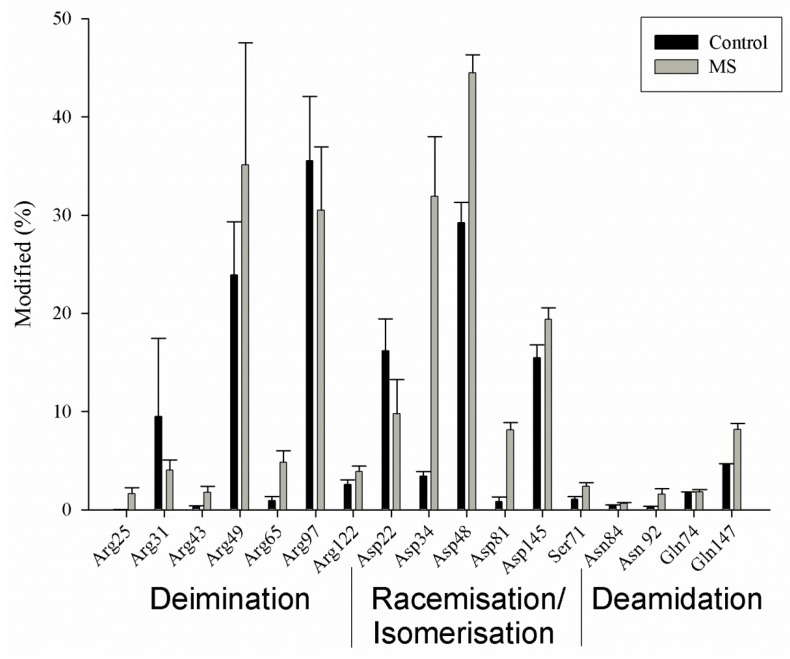
Myelin basic protein is highly modified in the adult human brain. Histogram showing the relative abundances of some of the major sites of modification in MBP from the cerebellum in controls and patients with MS. Three main types of modifications were observed: deimination of Arg (to form citrulline), racemisation and isomerisation of Asp, and deamidation of Asn and Gln. It should be noted that each site of Asn/Gln deamidation contains four Asp/Glu isomers due to the process of spontaneous racemisation and isomerisation (see Figure 1). The average ages of the two groups were controls 46 ± 22 years (*n* = 10) and MS patients 60 ± 12 years (*n* = 8). Shown are mean values ± SD. Detailed subject data from Friedrich et al., 2016 [9].

Proteomic analysis of the human cerebellum showed that MBP is highly modified in adults [9]. The extent of posttranslational modification is large, such that every region of the protein, showed some form of covalent alteration. In addition, these changes are quantitatively major (Figure 2). The covalent changes included deamidation of Asn and Gln residues, isomerisation of Asp, as well as deimination of Arg residues to citrulline. Some of these changes increased with age. Most were abundant even at age 18, which was the youngest sample available from the brain bank. That extensive MBP modification has taken place by such a young age may at first seem surprising, however the same phenomenon has been observed in the human lens. In the lens, the great majority of posttranslational modifications occur by age 20 [18]. Twenty years is, after all, equivalent to incubating a life-long protein at 37 °C for more than 175,000 h!

It should be noted that the PTMs of adult myelin discussed in this article take place on MBP that was significantly modified during development [21,22].

In accord with other long-lived proteins, one of the major changes characterised in adult human MBP was the formation of isoAsp residues. This form of Asp is quite different from the normal l-Asp that was present originally in the protein, and is due to the spontaneous formation of a cyclic intermediate [13]. At each site of isoAsp formation from l-Asp (or l-Asn), the polypeptide backbone has been extended by the insertion of a CH_2_ group (Figure 1). In reality, this particular modification leads to an even greater alteration in protein structure because a common product is d-isoAsp. In this case, the original l-amino acid site has been both extended and racemised. As well as creating novel antigens, protein unfolding will accompany these chemical transformations.

## 5. Myelin Basic Protein and MS

MBP from an adult, is highly heterogeneous and is a different protein from the MBP that was deposited originally in childhood. In one sense, an abundant new protein has been created in our brain. Every MBP polypeptide in cerebellar myelin contains isoAsp residues, as well as a number of citrulline residues [9] and each of these amino acids is novel. These structures are not found in normal proteins, including the original translated version of MBP.

It is not hard to imagine that in some cases the body may sense this ‘new’ form of MBP as being a foreign antigen and could commence an immune response. The precise cellular location of MBP is not yet clear. If it is partly extracellular then cellular degradation may not be required for immune priming. If MBP is an intracellular protein [23] then some cellular damage may be necessary. Not only does this provide an underlying mechanism for the autoimmune response, it also may explain why many cases of MS become evident in the fourth decade of life [24]. Even by the 30s, MBP has been almost totally transformed into a new protein. Although data on isoAsp formation and other PTMs in the MBP from children is lacking, it is likely that significant levels are present even in pre-teen years. This is because in other long-lived proteins (LLPs), such as lens proteins, racemisation occurs most rapidly in the first decade of life [18]. Therefore, the age-related changes to MBP and other myelin proteins could even play a role in the genesis of pediatric MS.

## 6. Myelin Basic Protein from MS Patients Is Different

**Figure 3:**
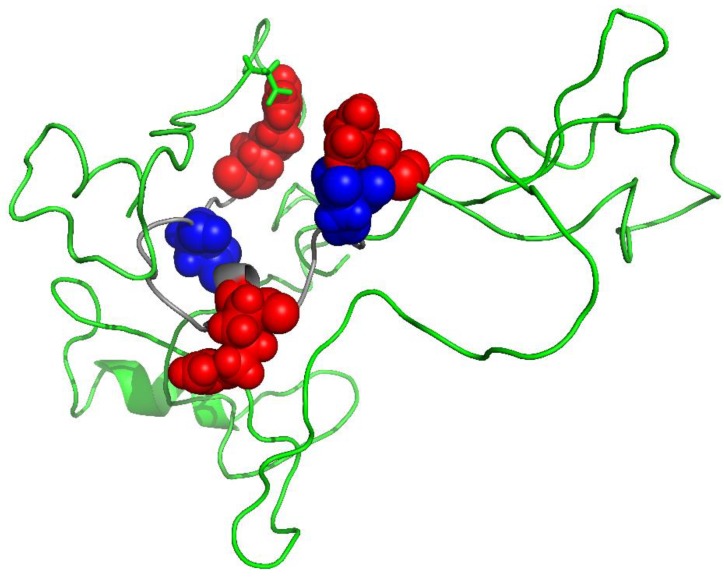
MBP in MS patients is different from MBP in controls. Highlighted in red (Arg) and blue (Asp) are 5 amino acid residues that differ significantly within a 19 amino acid sequence. This cluster includes Arg 31, Asp 34, Arg 43, Asp 48, and Arg 49. For each site, the degree of change is greater in the MS patients; the only exception being Arg 31. Statistical data are shown in Figure 2 and are summarised from Friedrich et al. [9]. The model of MBP was generated using I-Tasser [25] and PyMOL Molecular Graphics System was used to render the 3D structure.

If this ‘old protein’ hypothesis for the origin for MS is correct, why doesn’t everyone suffer from the consequences of such an untoward immune attack, since the brains of all adults are full of degraded MBP? One possibility is that the MBP from MS patients could be modified in a different way from the MBP in non-MS patients. Proteomic analysis showed that this was indeed the case. Although cerebellum samples from both normal and MS patients contained highly modified MBP, the MBP from MS patients was consistently different from the normal MBP at several sites (Figure 2). At each site, the degree of change was greater in MS patients; the only exception being Arg 31. In addition, once these MS-specific sites of modification were incorporated into a structure of MBP, they appeared to be localised into clusters (Figure 3).

One such cluster is a 19-residue sequence encompassing Arg 31 to Arg 49 (Figure 2 and Figure 3). This region includes Arg 31, Asp 34, Asp 48, Arg 43, and Arg 49 where in each instance, the extent of modification is significantly greater in MS patients. The single exception is Arg 31, where it is lower. Our hypothesis is that regions such as these may be particularly antigenic and could precipitate an immune response that leads ultimately to MS. There are precedents for each of the major modifications (i.e., isoAsp [9] and citrulline [26]) found to be more abundant within the MBP clusters from MS patients, acting as potent antigens. In MBP from MS patients both of these immune promoters are found close together in space [9] so there could well be a synergistic interaction of these novel amino acids. In effect, aged MBP from MS patients may be ‘more antigenic’ than aged MBP of unaffected individuals. It should be noted that other proteomic investigations have also detected differences (e.g., in deamidation) between the MBP of control and MS patients [26]. These proteomic data are bolstered by studies that have reported that myelin from MS patients differs from controls e.g., in being ‘developmentally immature’ [27].

## 7. Considerations If MS Results from Age-Related Changes to MBP

There are a number of consequences that flow from the discovery that MBP is highly modified with age and that some changes to MBP are characteristic of MS.

Firstly, if indeed human MS is an autoimmune disease provoked by an endogenously-formed antigen, what, if any, biological processes exist to modulate the response? Can some protein modifications be tolerated? After all, despite MBP being highly modified in all adults, only a small fraction of people suffer disease symptoms.

To what extent does ongoing (in the adult) immune tolerance play a role in the body where there is, in effect, a failure to respond to the formation of a major antigen that was absent at birth? Foxp3 + T regulatory cells are likely to play an important role in this modulation. In addition, there are several chaperone proteins present in the CNS (e.g., alpha crystallin) that can bind to modified, unfolded proteins and in this way could cover potentially antigenic sites before they are recognised by the immune surveillance machinery. Could this putative protective system be less effective in people who go on to develop MS?

As with immunisation, the presence of a novel antigen alone may be insufficient to trigger a full immune response. An adjuvant is necessary. Another immune-promoting event (e.g., an infection) may be necessary for a complete response which ultimately leads to MS. In this regard, it is interesting to note that bacterial and viral proteins contain unnatural amino acids such as d-isoAsp and d-isoGlu, so molecular mimicry could play a role. It is known that there is a link between infection and MS [28] and the presence of predisposing genes is also likely to be implicated.

**Figure 4:**
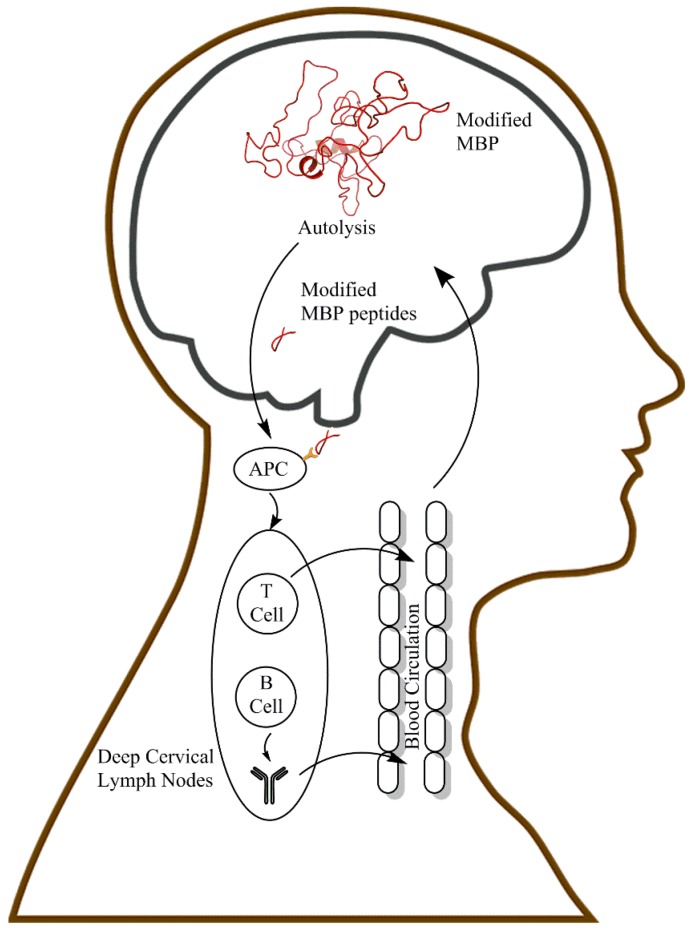
A simplified scheme illustrating how modified MBP in the brain could lead to the induction of MS. One mechanism that could underpin the onset of MS is illustrated. MBP in the human CNS is heavily modified by age 18, with some modification sites being MS-specific. Modified MBP and/or peptides derived from it by autolysis [14,35], or possibly protease action, diffuse via the lymphatic system to the CSF. Subsequent drainage via the lymphatic vessels to the deep cervical lymph node may permit priming of T cell responses. Once primed, T and B cells become self-reactive. In ways analogous to those described for EAE, these self-reactive species cause damage to the myelin and inflammation of the CNS, which manifests as MS (see text for details). APC, Antigen-presenting cell.

T cells have long been thought to be implicated in MS pathogenesis, in particular the inflammation associated with the condition. Recent studies have also highlighted the important contribution of B cells. Evidence for a major role of B cells in MS is suggested by the clinical benefit derived by peripheral B cell depletion using drugs such as rituximab, an anti-CD20 agent [29]. A possible way in which modified MBP (and other myelin proteins) may elicit an immune response is depicted in Figure 4.

It is not yet known how normal individuals are able to maintain immunological tolerance towards a new self-antigen (i.e., modified MBP) that is present in large amounts in the adult brain. As mentioned above, there are some intriguing observations that suggest a role for peripheral B cell depletion in this process. In an elegant study to determine whether B cell tolerance checkpoints are defective in MS patients, it was discovered that most suffer from impaired peripheral B cell tolerance, resulting in the accumulation of large numbers of autoreactive and polyreactive mature naive B cells, which express white matter–reactive antibodies [30]. Aspects of B cell biology [31] and T cell interactions with respect to MS have been more extensively reviewed elsewhere [32]. Interestingly, in surgical brain injury, it has been found that immune tolerance can be induced by hepatic portal vein and intrathymic injection of MBP [33]. Possibly, in individuals who are not affected by MS, continuous exposure to low levels of modified MBP via the route depicted in Figure 4 may act in a similar manner to therapeutic administration of a causative antigen as outlined by Verhagen et al. [34]. In theory, if such a mechanism were impaired or disrupted, exposure to modified MBP may trigger MS.

## 8. Gender, MS, and Age-Related Changes to MBP

**Figure 5:**
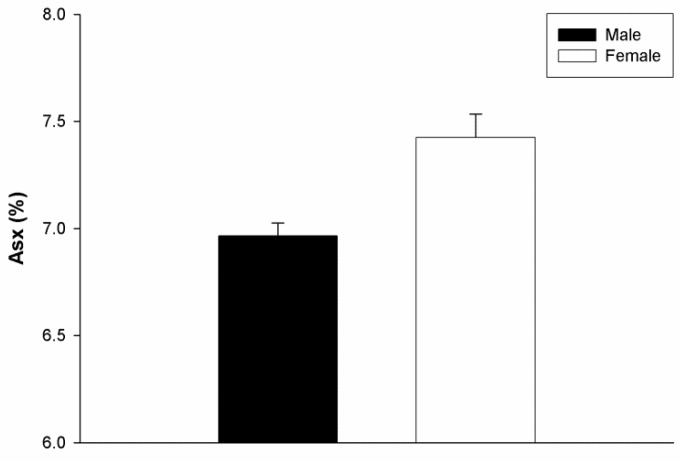
MBP is more highly racemized in females. Racemisation of aspartic acid + asparagine (Asx) in the MBP of age-matched male (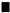) and female (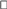) subjects. Elevated levels of d-Asx were found in females by comparison to males (*p* = 0.029, Mann–Whitney U test). Male *n* = 4, female *n* = 4, age range 51–87 years. Mean ± SEM.

It is well known that MS affects more females than males. If MS can indeed be ascribed to the body’s response to myelin deterioration in the brain, then it may be predicted that women could harbour a greater degree of protein modification than men. Remarkably this has been observed for MBP (Figure 5). The numbers analysed thus far are small, however the findings are in agreement with a separate study of human MBP that showed higher levels of Asn deamidation, Gln deamidation, and Arg deimination in women than men [35].

## 9. Linkage between the CNS and the Periphery in MS

One reason for believing that an immune response is involved in the induction of MS comes from animal studies. EAE is a widely studied animal model of human central nervous system (CNS) demyelinating diseases, such as multiple sclerosis [36]. EAE can be induced in a number of species and the most commonly used antigens for injection are spinal cord homogenates, myelin proteins such as MBP, proteolipid protein (PLP) and myelin oligodendrocyte glycoprotein (MOG), as well as peptides derived from these proteins. Each results in a model with slightly different disease characteristics regarding both immunology and pathology. EAE can also be induced by the passive transfer of T cells reactive to these myelin antigens [37].

EAE demonstrates that once the body has been primed by exposure to a CNS antigen, that it can respond with an attack on the brain. As has been recently reviewed by Louveau et al. [38] the CNS, which was historically viewed as being immune privileged, is now recognized to be in continuous communication with the rest of the body. One way in which an immune response to degraded MBP could be initiated, is via the “glymphatic” system involving deep cervical lymph nodes [39]. The finding that MBP is long-lived, also provides a mechanism for how modified peptides derived from it might travel from their source in the CNS to sites in the periphery where an immune response could be initiated. Spontaneous cleavage of long-lived proteins at Ser [40] and Asn [13] could lead to the release of modified MBP peptides from myelin into the cerebrospinal fluid (CSF) (Figure 4).

The various T cell responses in the CNS are controlled in a complex manner as summarised in a recent comprehensive review [41] and structural organisation of the CNS plays a key role in determining the details of the overall immune response [36]. Evidence in support of the scheme outlined in Figure 4 is derived from several sources. For example, induction of apoptosis in oligodendrocytes is accompanied by the accumulation of phagocytes containing myelin antigens in the lymph nodes that drain the CNS [42]. In addition, oligodendrocyte death can cause CNS antigens to drain from the CNS in amounts sufficient to induce a systemic T cell response against MOG [43].

## 10. Autoantibodies and MS

There is compelling evidence from animal model systems that myelin antigens can act as drivers for the induction of MS, and if this were true, it might be expected that MS patients display higher levels of anti-myelin antibodies than unaffected individuals. The results of human antibody studies in MS patients and controls have often been mixed [44,45]. The proteomic results may provide one reason for this lack of consistency. Based on the proteomic data from human cerebellum, there is probably very little unmodified MBP left in normal adult brain. The degree of MBP modification is simply so great. Preliminary data suggest that MBP from other brain regions is also highly modified. Therefore, screening for antibodies to the MBP polypeptide as it was originally translated (and as it once existed in childhood), may not be an appropriate method to assess whether MS patients have clinically-relevant autoantibodies to adult myelin proteins.

Given the most recent proteomic data [9,26] that has uncovered brain proteins that are highly altered in adults, a better question may be: are antibodies that recognise highly-modified MBP present in MS patients? Antibodies may cross-react to varying degrees with the original unmodified MBP, or possibly may not recognise it at all. Now that MS-specific sites on MBP have been characterised [9,26], a search for antibodies to these particular sites in MS patients should be a priority. In other words, using relevant antigens for antibody screens may be important. Unmodified (original) MBP may not be the best choice and this factor could be one explanation for variability in the published results of serum screening of MS patients for antibodies to MBP [44,45]. Our preliminary data indicate that the other long-lived proteins in myelin (e.g., PLP and MOG [7]) have also been modified over time and could potentially become new self-antigens, which have the potential to trigger autoimmunity. The same caveats with regard to the screening for MS-relevant autoantibodies (and T cells) to these proteins as outlined for MBP, may apply to them as well.

## 11. Cellular Aspects of MS

B and T cell responses to self-antigens are clearly important and mechanisms of central tolerance for B cells have been reviewed [46]. Significant increases in the number of autoreactive B cells have been observed in MS patients [47]. In general, it appears that B cell responses to single sites of PTM in self-antigens are likely to be promiscuous, in the sense that the antibodies recognise both modified and unmodified forms of the self-protein. Presumably this is because the amino acid sequences on either side of the modification remain the same. On the other hand, T cell responses tend to be more specific to the site of modification [48]. In the context of a B cell response in MS, it is important to recognise that MBP has been modified at many sites [9].

Another hypothesis is that the modified MBP could act as a trigger of the innate immune response by binding to pattern recognition receptors and/or damage-associated molecular pattern receptors to drive inflammatory processes and lower activation thresholds of T and B cells.

## 12. A New Theory for MS

It is a corollary that if the extensive modification of MBP in the CNS, coupled with its drainage into the periphery and the consequent induction of an immune response, (as illustrated in Figure 4), is validated, it changes the way in which MS is viewed. For example, it renders largely unnecessary a widely held hypothesis for the induction of MS, based on the sensitisation of autoreactive T cells by non-CNS antigens derived from the periphery (e.g., molecular mimicry). In the hypothesis outlined in this article, MS is triggered primarily by the formation of a novel endogenous antigen rather than by the body’s altered response to an existing antigen.

## 13. Conclusions

With regard to the consequences, perhaps the most important for MS is that the existence of MS-specific sites on a major myelin protein opens up the possibility of targeted drug design to prevent, or treat, MS. Although it is not yet clear why MS presents in several forms, if MS does result from an immune response to particular modified sites on MBP, then compounds that specifically recognise and mask these sites could potentially offer a degree of protection. This may be the dawn of a new era in the treatment of MS.
